# Use of Protein Repellents to Enhance the Antimicrobial Functionality of Quaternary Ammonium Containing Dental Materials

**DOI:** 10.3390/jfb11030054

**Published:** 2020-08-01

**Authors:** Leopoldo Torres Jr, Diane R. Bienek

**Affiliations:** ADA Science & Research Institute, LLC, Innovative & Technology Research, Frederick, MD 21704, USA; torresl@ada.org

**Keywords:** protein repellent, restorations, zwitterionic polymers, dental materials, antimicrobial, antifouling

## Abstract

An advancement in preventing secondary caries has been the incorporation of quaternary ammonium containing (QAC) compounds into a composite resin mixture. The permanent positive charge on the monomers allows for electrostatic-based killing of bacteria. Spontaneous adsorption of salivary proteins onto restorations dampens the antimicrobial capabilities of QAC compounds. Protein-repellent monomers can work with QAC restorations to achieve the technology’s full potential. We discuss the theory behind macromolecular adsorption, direct and indirect characterization methods, and advances of protein repellent dental materials. The translation of protein adsorption to microbial colonization is covered, and the concerns and fallbacks of the state-of-the-art protein-resistant monomers are addressed. Last, we present new and exciting avenues for protein repellent monomer design that have yet to be explored in dental materials.

## 1. Introduction

Tooth decay, also known as dental caries, is one of the most prevalent infections globally that afflicts both the developed and developing world. It affects young and old at large percentages, while being a preventable disease. Due to the high incidence of patient affliction, the economic toll is large in the US, approximately $442 billion [1]. To combat caries, clinicians remove the decayed tissue and replace with restorative materials. These composite materials consist of a stiff polymeric matrix (e.g., bisphenol A-glycidyl methacrylate (BisGMA) [2], urethane dimethacrylate [3], or methacryl polyhedral oligomeric silsesquioxane [4,5]) and inorganic filler components (i.e., amorphous calcium phosphate nanoparticles [6], borosilicate microparticles [7], and hydroxyapatite [8]). Additional problems arise from secondary caries, subsequent infections beneath or in the micro-cracks of the composite [9]. This occurs up to 44% for all adult patients and could be mitigated by antimicrobial technologies [10].

Integration of polymerizable antimicrobial methacrylates in dental resin offers the benefit of providing lasting antimicrobial activity, while being chemically stable. Specifically, quaternary ammonium containing (QAC) monomers have been incorporated into dental resins, to enable contact-killing of microorganisms. This concept was first introduced in 1993 by Imazato et al. [11]. Briefly, a quaternary ammonium compound, 12-methacryloyloxydodecylpyridinium bromide (MDPB), was incorporated into a resin, to formulate an antimicrobial composite [12]. Reports have indicated that QAC compounds destroy bacterial cell membrane integrity and eventually lead to cell death [13,14,15]. The design of these monomers has been heavily studied to optimize antimicrobial capabilities and elucidate the mechanism of bacterial killing [16]. For example, Li and coworkers synthesized QAC monomers with varying carbon lengths, following the positive quaternary amine, to enhance the insertion of the dangling monomer into the *Streptococcus mutans* membrane [17]. Another strategy has been to investigate monomers with varying degrees of flexibility, for improved incorporation into the bacterial membrane. The effect of alkyl chain length on antimicrobial properties of monomethacrylate monomers suggested a rise in antibacterial activity with the increasing alkyl chain [18,19]. However, the antimicrobial efficacy–structure relationships are not strictly linear. For instance, the longer chain length of novel adhesive methacrylate dental monomers had a less marked effect on reducing *S. mutans* biofilms [20]. Furthermore, when drawing conclusions about chain length, it is important to consider that antimicrobial functionality is also affected by molecular mass, spacer rigidity, hydrophobicity, charge density, and charge distribution [21,22]. Since the first Imazato and coworkers’ QAC resin manuscript, advances have been made in synthesizing QAC monomers with dual functionality, such as increased shear bond strength [20,23,24] and silane-coupling capabilities [25,26,27]. Similarly, other antimicrobial approaches have been studied in dentistry, including metallic nanoparticles with inherent bactericidal properties [28] and are not discussed in this review.

While previous studies on QAC dental materials are significant, their impact has been dampened by the reduction in antimicrobial efficacy due to protein adsorption [29]. Salivary proteins form a thin coating onto the enamel surface, called the pellicle [30]. The pellicle allows for the attachment of early colonizing bacteria [31]. Many bacterial species possess surface structures (i.e., fimbriae and fibrils), which facilitate their attachment [32]. In the early stages of biofilm formation, planktonic bacteria directly attach to surfaces or indirectly bind to other bacteria that have already colonized [33]. Acidic and high-molecular-weight mucin fractions, acidic proline-rich proteins, and a multidomain glycosylated protein of the salivary pellicle are reported to bind bacteria and adhere to the non-native surface, to support biofilm attachment at the composite–adhesive–tooth interfaces [34]. The proteins and pathogens (and their interactions) attributing to failure at the composite–tooth interfaces have been presented in an elegant review [35]. After attachment, the bacterial cells proliferate, form microcolonies, and mature. Generally, biofilms can be morphologically heterogeneous 3D structures with the shape affected by spatiotemporal stress [36]. The 3D biofilm structures can be interspersed with bacteria-free channels used as diffusion pathways [32]. Dental caries and periodontal disease are a net result of the cross-talk between pathogenic dental-plaque biofilm and the host-tissue response [36]. While clinical examination and X-rays are commonly used to diagnose oral disease, advancement of salivary biomarkers and metaproteomic analyses of the oral microbiota may be exploited for future diagnosis of opportunistic and infectious disease [37].

**Figure 1:**
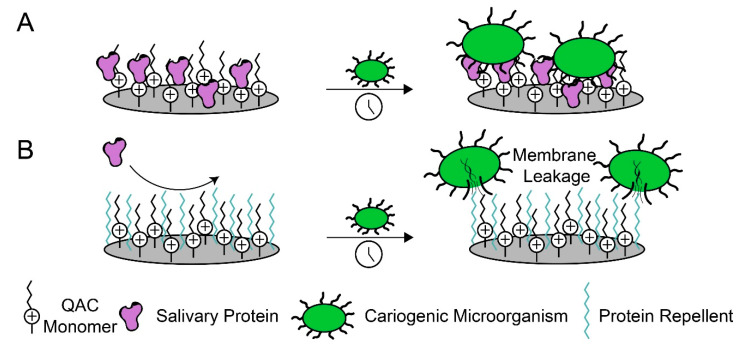
Dual functional dental materials enable contact-killing of cariogenic microorganisms by repelling proteins and disrupting bacterial membranes via charged interactions. (**A**) Salivary proteins adsorb to quaternary ammonium-containing (QAC) monomers, inhibiting their long-term antimicrobial properties. (**B**) Protein-repellent molecules work with QAC monomers to disrupt the formation of biofilms.

To improve the antimicrobial properties of QAC monomers, protein-repellent functionality should be incorporated to prevent the extent of adsorption that current restorations experience (Figure 1). To accomplish this, the dental-material community has implemented approaches from the surface-science and blood-contacting material literature. Much of the recent literature has focused on 2-methacryloyloxyethyl phosphorylcholine (MPC), a commercially available and U.S. Food and Drug Administration-cleared zwitterionic polymer with well-studied protein-repellent capability.

The objective of this review is to focus on (1) the theoretical and practical considerations of protein adsorption; (2) methods to quantify protein adsorption; and (3) protein repelling functionality in dental restoratives and mouthwash technologies. Moreover, we also identify challenges with commonly used protein repellants and consider the potential of developing novel monomers.

## 2. Theoretical and Practical Considerations for Protein Adsorption

Protein adsorption is a spontaneous process arising from a contribution of electrostatic and hydrophobic interactions, or hydrogen bonding [38]. Net charges on proteins can electrostatically bond with surfaces that are oppositely charged. This process can be reversed as the pH is altered, owing to the proteins pKa [39]. Additionally, hydrophobic regions of proteins can unravel (face outward) to bond with hydrophobic surfaces, minimizing the interactions between water and salivary proteins and between the surface and salivary proteins [40]. Lastly, both proteins and dental materials can participate in hydrogen bonding with each other if that is more favorable than surface solvation interactions [41].

Understanding the mechanisms of adsorption has led to the development of design principles for protein-repellent materials. The Whitesides group in 1993 reported a method for fabricating self-assembled monolayers (SAMs) as a tool to study protein adsorption [42]. These SAMs consisted of densely packed alkylthiol molecules that aligned parallel with one another on the surface of gold substrates. The tail end of the alkylthiol molecules were terminated with a functional group that could be covalently bonded to a chemical group of interest. Surface Plasmon Resonance (SPR) was coupled with a SAMs device to quantify the adsorption, observed as a change in frequency of the plasmon resonance. Their group subsequently followed their initial article with a survey of chemical structure relationship on adsorption, for which they reported four distinct rules for effective protein-repellent surfaces: The surface exposed monomers should be (1) hydrophilic; (2) contain hydrogen bond acceptors; (3) have no hydrogen bond donors; and (4) be electrically neutral [43,44,45]. Poly(ethylene glycol) (PEG)-based polymers [46] and many zwitterions [47] fall into this set of criteria and have been widely used for coatings in blood-contacting materials and implants.

In practice, additional considerations need to be taken into account to ensure effective protein repelling. The findings of the Whitesides’ group can only translate if a critical density of antifouling monomers is on the surface of the material. Surface-coating treatment of protein-repellent monomers will not be as effective in dentistry, due to the formation of micro-cracks in composite materials produced during polymerization shrinkage [3]. These cracks are susceptible to bacterial colonization and consequently secondary caries, which are largely responsible for the failure of composite fillings [10]. It is imperative that dental materials contain protein-repellent molecules on their surface and in the bulk without (or minor) effect on the mechanical properties. In the same vein, researchers have developed hydrogels with protein-repellent monomers incorporated in the bulk and surface [48,49,50]. Last, the QAC monomers have functional groups that inherently adsorb proteins more readily. Specifically, the charged quaternary amine will participate in electrostatic interactions with proteins, leading toward adsorption. The long alkyl chain in many QAC monomers will also aid in hydrophobic–hydrophobic interactions, causing unfavorable adsorption. Therefore, dental materials require sufficient coverage of protein-repellent monomers to minimize attractive forces between proteins and QAC monomers.

## 3. Characterization Methods for Quantifying Protein Adsorption

**Figure 2:**
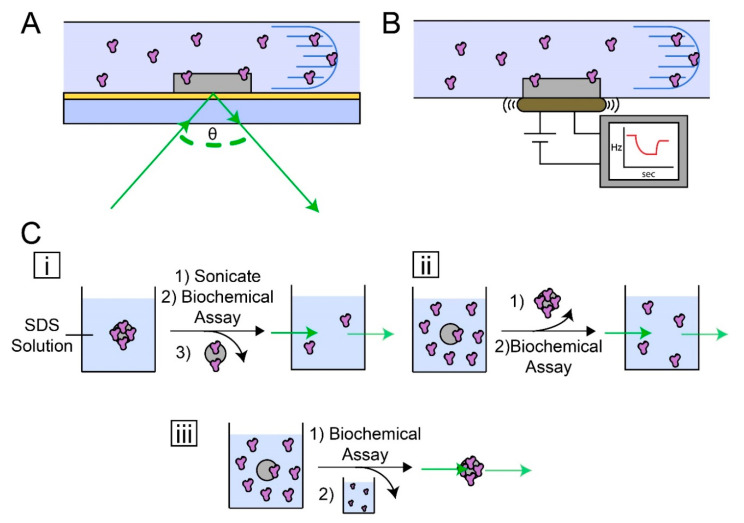
Characterization methods for quantifying protein adsorption on dental materials. (**A**) A general Surface Plasmon Resonance (SPR) device setup. Protein solution is flowed through a microfluidic channel and onto the material of interest bonded to a thin gold layer. Light is projected through a prism and onto the gold layer to discern protein–material interactions. (**B**) A general quartz crystal microbalance (QCM) device setup. The material of interest is fabricated onto a piezoelectric sensor. As protein accumulates onto the material, the vibration frequency changes. (**C**) Colorimetric methods for quantifying protein adsorption. (**i**) A disk with protein adsorbed to the surface is placed into a sodium dodecyl sulfate (SDS) buffer, to remove the protein. This solution is then analyzed by using an amino acid colorimetric reactive dye. (**ii**) A material of interest is placed in a protein solution of known concentration. After some time, the material is removed, and the remaining solution is analyzed. (**iii**) A material is placed in protein solution and is removed from the solution after a desired time point. The material with adsorbed protein is placed in a solution with colorimetric reagents, and the optical density is measured.

Several techniques and assays have been developed to study the degree of protein coating on material surfaces (Figure 2). These methods range in sensitivity and each have trade-offs and should be considered for studying the adsorption of protein on dental materials. A comprehensive guide to characterization techniques for protein adsorption can be found in a previous article [51].

The most sensitive techniques (able to detect 1 ng to 1 µg of protein) commonly utilized are SPR or quartz crystal microbalance (QCM). Both analyze the surface of a small (<5 × 5 mm) substrate that is functionalized with a protein repellent of interest. Samples are prepared by coating or chemically functionalizing the surface with the protein repellent of interest. By preparing a thin sample, the interactions between a protein solution and protein-repellent monomer can be probed. In a typical SPR detection apparatus, a thin gold-coated slide is coated with the monomer of interest [42,52]. The slide is mounted, glass side down, onto a prism, and the functionalized side is used as part of a microfluidic channel. A light source with narrow range emission is projected through the prism and glass slide and reflected off the thin gold layer. A detector collects the angle of the reflected light, which corresponds to the index of refraction of the functionalized gold layer. When a protein solution flows onto the functionalized surface, the index of refraction increases, causing the angle of the reflected light to change. This change can then be used to calculate the mass of protein on the surface of the substrate.

QCM devices implement a piezoelectric functionalized substrate [53,54,55]. When a current is applied to the substrate, the material vibrates at a frequency proportional to its mass. The piezoelectric material surface is coated or chemically bonded with a material of interest, to probe how much protein adsorbs to the material surface. These substrates are incorporated into microfluidic devices that flow protein solutions onto the substrate surface and are coupled with real-time sensing. When proteins adsorb onto the substrate, the frequency at which it vibrates changes. This change in frequency is converted to the mass of protein adsorbed to the substrate. QCM can be used to yield dynamic properties of adsorbed proteins, such as revealing changes to the salivary pellicle on hydroxyapatite surfaces when various detergents are flowed over the substrates [56]. In addition to monitoring protein adsorption, QCM has been used to probe the formation of biofilms and bacterial death in a clinically relevant microorganism model [57]. SPR and QCM techniques are useful for protein interactions with high-density surfaces and are best for studying low adsorption, as they saturate with milligram quantities of protein.

Dental composite materials exhibit polymerization stress, causing the composite to crack when curing [3]. These cracks expose the bulk, leaving sites for adsorption without a surface-modified layer. A more practical method for detection of protein adsorption is through colorimetric analysis of protein solution surrounding a dental material. Biochemical assays such as the bicinchoninic acid (BCA), Lowry protein, or Bradford assay utilize reagents that alter their visible-light absorbance upon reacting with a protein in solution. In this regard, three protocol variations have been widely used by the dental materials community. A material sample is submerged in a protein solution for a predetermined time. (1) The material is rinsed with saline solution, to remove non-adsorbed proteins, and the adsorbed protein is removed by rinsing the material with a sodium dodecyl sulfate (SDS) solution. This SDS solution is then used in a colorimetric assay [58]. While this method is the most used in the dental material literature, a comprehensive investigation determined that SDS rinsing does not adequately remove adsorbed proteins [59]. This may lead researchers to conclude a protein-repelling capacity that is inaccurate. (2) In lieu of rinsing the material with SDS to remove the adsorbed protein, the protein solution and material can be vortexed to remove non-adsorbed proteins, and the protein solution surrounding the material is analyzed. This method yields quantification of the remaining solution compared to the initial starting concentration. (3) A small sample of protein solution is placed on a flat, clear, and polymerized dental resin. After the desired time, the non-adherent protein is removed by rinsing with saline. The material is then submerged in the reagents of a colorimetric assay, allowing the adsorbed proteins to react with the assay reagents. The optical density at the assays absorbance wavelength is then performed to quantify the adsorbed proteins [60,61].

Topographical features and visualization of adsorption are important in understanding the growth of biofilm formation on dental materials. Looking toward the future, dental materials researchers should explore atomic force microscopy as a characterization tool. This technique probes the surface of materials, using a cantilever tip (100 nm–100 µm) and a laser to gauge the position of the tip, producing geometric information of a material [62]. Few dental material groups have explored the use of atomic force microscopy. to visualize protein adsorption on the surface [63]. Information that could be useful to researchers include the homogeneity of the adsorption layer, thickness of the adsorption layer, and force required to break bonds between the material surface and adsorbed proteins.

## 4. Dental Materials with Protein-Repellent Functionality

MPC is a methacrylate zwitterionic polymer that contains a negatively charged phosphorylcholine and a positively charged quaternary ammonium head. It is the most investigated molecule for protein-repelling dental materials [47,64,65]. It blends well into BisGMA/triethylene glycol methacrylate (TEGMA) and other hydrophilic resins. It was first introduced in the dental material literature by the Xu group in 2015 as an additive to a 50:50 BisGMA and TEGMA (BT) resin with dimethylaminohexyadecyl methacrylate (DMAHDM) and barium boroaluminosilicate filler [58]. The authors found that, out of various compositions investigated, a 3% (wt/wt) MPC and 1.5% (wt/wt) DMAHDM composite demonstrated nine times less bovine serum albumin (BSA) adsorption (~1 µg/cm^2^), compared to the commercial control. For this composition, the flexural strength decreased from 100 to 80 MPa and elastic modulus from 6.7 to 6.5 MPa, compared to the resin without MPC. Oral biofilms, derived from the saliva of human donors, were cultured on the composites, to assess the antimicrobial capabilities of the MPC composites, with and without DMAHDM. Compared to the commercial control, a 3% MPC composite exhibited an order of magnitude lower total microorganisms, while the 3% MPC + 1.5% DMAHDM exhibited a three order of magnitude decrease of total microorganisms. This result demonstrated that the full potential of QAC resins could be realized with the addition of MPC.

The Xu lab continued their efforts into investigating the capability of MPC in many facets of materials in dentistry. A subsequent article optimized the addition of MPC into BT resins to identify a formulation with high protein repellency, without compromising the mechanical properties of the material [66]. They demonstrated that the flexural strength and elastic modulus suffer with materials containing 4.5% MPC and above. Specifically, the 4.5% MPC composite exhibited a flexural strength decrease to ~60 MPa compared to ~85 MPa in the case of the control. Moreover, the elastic modulus decreased to 5 MPa, compared to 6 MPa, as it was with the control. In a BSA adsorption assay, the researchers reported that a 3% MPC sample decreased the amount adsorbed by 85%, as compared to a polymerized BT sample. This same formulation also exhibited eight times fewer total microorganisms compared to the BT control.

MPC was then incorporated into a dental primer, to establish whether it could be useful as a restoration [67]. In conjunction with DMAHDM, MPC mixed into a commercial dental bond primer (3M Scotchbond Multi-Purpose Adhesive and Primer). A 7.5% MPC composite demonstrated less than 1 µg/cm^2^ of protein adsorption, nearly 10 times less than the control. The same formulation had a comparable dentin shear bond strength, at ~27 MPa, compared to the control, at ~33 MPa. The degree of conversion was minimally impacted in all the formulations tested, indicating that MPC blends well with conventional dental resins. Last, the 7.5% MPC formulation was the most effective at reducing the total microbial count, by four orders of magnitude lower than the primer control. A follow-up investigation with amorphous calcium phosphate nanoparticles as the filler revealed that the shear bond strength decreased from 30 to 22 MPa [68]. The addition of the particles did decrease the shear bond strength to 25 from 30 MPa for a filler content of 30%, but did not alter the protein-repelling effects or antimicrobial efficacy. Even in long-term water aging of 180 days, MPC composites demonstrated closely similar protein-repelling and antimicrobial efficacy, likely due to the high degree of conversion [69].

To probe whether MPC inhibited or enhanced the release of calcium and phosphate ions from ACP particles, a 1:1 mixture of ethoxylated bisphenol A dimethacrylate (EBPADMA) and pyromellitic dianhydride glycerol dimetrocralte (PMGDM) was used as the resin matrix, abbreviated to EBPM [70]. This resin formulation was found to allow for the release of calcium and phosphorous ions when used with a ACP particle filler [71]. The combination of resin, MPC, and DMAHDM did not alter the protein-repelling properties of a 3% MPC formulation. In a four-organism biofilm challenge, a 3% MPC + 3% DMAHDM composite inhibited the colony count of *Porphyromonas gingivalis* and *Aggregatibacter actinomycetemcomitans* by four orders of magnitude and *Prevotella intermedia* and *Fusobacterium nucleatum* by three orders of magnitude, as compared to the resin alone [70]. The hydrophilicity of MPC caused more swelling in the composites, leading to a higher release of calcium and phosphorous ions, compared to the formulation without MPC [72]. By altering the amount of MPC, the amount of ions released could be tuned [73].

Poly(methyl methacrylate) (PMMA) is a common biomaterial routinely used in dentures and can be a breeding ground for oral microbes due to the heavy coating of salivary proteins it endures. MPC was incorporated into a methyl methacrylate monomer mixture and thermally cured. A 3% MPC formulation was enough to substantially decrease the amount of BSA adsorbed to ~2.0 µg/cm^2^ compared to bare PMMA (~12 µg/cm^2^) [74]. Computational modeling of surface interactions of MPC grafted onto PMMA revealed that MPC forms a tight hydration layer and a network of hydrogen bonding between adjacent MPC chains (in high-density grafting), which inhibits the adsorption of proteins and the anchoring of bacteria to the material surface [75].

More recently, there has been an effort to understand how QAC composites affect the microbial composition in biofilm models. MPC in QAC composites were shown to be more effective at decreasing the microbial growth of a single species biofilm of *P. gingivalis* [76]. As the microbial diversity increased, the composite was less effective at decreasing growth. Ultimately, the composite decreased the total microbe count by three orders of magnitude, compared to the resin control. It is well-documented that genetic information is shared between microbes in biofilm communities that act as a defense against chemical agents [77]. These composites also have shown to decrease the *S. mutans* composition in biofilms, leaving non-cariogenic species to thrive [78]. A summary of the protein repellent dental material capabilities discussed can be found in Table 1.

## 5. Mouthwash Coating Technology

A potential solution to repetitive cariogenic bacterial attachment to dental tissue is through the use of oral rinses with safe protein-repellent molecules that bind to enamel, root, or dentin surfaces. Recently, this concept was tested in a small clinical study by evaluating the number of microbes in dental plaque before and after rinsing with a solution of 5% MPC in saline [79]. Twenty patients had oral samples collected via gargle immediately after and 5 h after brushing their teeth. Half of the subjects were given a saline rinse as a control and the other half the MPC treatment. The patients treated with MPC saw a microbial decrease of 45%, compared to the control, through electric counting of the patients’ gargle, indicating that protein adsorption was lessened. The number of fusobacteria, a mediator of bacterial aggregation and plague formation, was inhibited by this treatment. While no chemical modification of the oral environment was mentioned, a more biologically compatible material may be necessary for frequent rinses. In a separate study, a self-assembly approach was used to coat the oral cavity with lysozyme aggregated particles tethered to PEG to repel proteins [53]. Lysozyme was reduced with tris(2-carboxyethyl)phosphine (TCEP) to induce aggregation. These particles have a high tendency to physically bond to many material surfaces, including dentin and enamel surfaces. The particles densely pack at the surface, allowing for the formation of a tight hydration shell around the outward facing PEG molecules, yielding an effective protein repellent monolayer. The authors successfully tested their coatings against BSA, concanavalin A, fibronectin, saliva, fetal bovine serum, milk, egg whites, and various polysaccharides. Importantly, the authors found they can overcome the potential of esterase degradation by incorporating both positive and negative charges into the PEG molecules to induce a zwitterion effect and increase protein repellency of esterases. These approaches are novel and justify further investigations to determine feasibility and effect on the biofilm formation.

**Table 1:** Summary of bovine serum albumin (BSA) adsorption values for references in this review.

Protein Repellent Compound	Bulk Material	Filler	Adsorption Value (ng/cm^2^)	Quantification Method	Reference
3% MPC (w/w)	25.5% 1:1 BisGMA/TEGDMA	70% Barium boroaluminosilicate	1240	SDS removal + BCA Assay	[58]
3% MPC	27% 1:1 BisGMA/TEGDMA	70% Barium boroaluminosilicate	960	SDS removal + BCA Assay	[66]
7.5% MPC	75% 1:1 Scotchbond Multi-Purpose Primer and Adhesive	15% Amorphous calcium phosphate	321	SDS removal + BCA Assay	[68]
3% MPC	25.5% 50:50 BisGMA/TEGDMA	70% Barium boroaluminosilicate	972 (with 180 days water aging)	SDS removal + BCA Assay	[69]
3% MPC	24% 1:1 EBPM	20% Amorphous calcium phosphate; 50% barium boroaluminosilicate	1200	SDS removal + BCA Assay	[70]
3% MPC	44.5% PMGDM, 39.5% EBPADMA, 10% 2-hydroxyethyl methacrylate, 5% BisGMA	30% Amorphous calcium phosphate	416	SDS removal + BCA Assay	[72]
3% MPC	47.75% Nature Cryl^TM^ liquid	47.75% Nature Cryl^TM^ powder	2150	SDS removal + BCA Assay	[74]
3% MPC	24% 1:1 EBPM	20% NACP, 50% barium boroaluminosilicate	1000	SDS removal + BCA Assay	[76]
PEG	Self-assembled PEG lysozyme	N/A	8	QCM	[53]
33% trimethylamine *N*-oxide Zwitterionic Hydrogel	N/A	N/A	3 *	SPR	[80]
9% Poly(carboxybetaine acrylamide) Zwitterionic Hydrogel	N/A	N/A	4.3 *	SPR	[49]

* Adsorption values for human serum, not BSA.

## 6. Limitations of Existing Technologies

While MPC has gained attention in the dental material literature, it has several shortcomings: (1) MPC contains an ester group connecting the zwitterionic component and the polymerizable methacrylate group. It is well-documented that ester bonds can be cleaved by esterases in saliva via hydrolysis [81]. (2) To achieve low levels of protein adsorption (ng/cm^2^), MPC concentrations ≥ 5% need to be included into the bulk, which has a detrimental effect on the flexural strength, elastic modulus, and hardness. (3) Currently, there is a paucity of information regarding the long-term protein adsorption of dental resins containing MPC [82]. (4) Although, in theory, MPC meets many design criteria for being the superior protein-repellent candidate, many groups have shown that other conventional polymers outperform MPC in various experimental models [83,84,85,86]. New zwitterionic polymers are needed to overcome some of the long-term concerns with MPC. In addition, the potential clinical benefit needs to be confirmed, as MPC may have a deleterious effect on the remineralization capacity of restoratives (i.e., possible binding of the re-mineralizing calcium ions by MPC).

Recently, a zwitterionic polymer was synthesized by oxidizing an acrylamide monomer with 50% hydrogen peroxide, resulting in a permanently positive quaternary amine bonded to a permanently negative oxygen atom [80]. The proximity of the two charged atoms forms a tight hydration layer in an aqueous environment, leading to high repellent efficacy (5 ng/cm^2^ by SPR). The polymer also exhibited satisfactory cytotoxicity and immunogenicity in a mouse model and should be studied as a candidate for protein repellency in dental materials. A separate group also synthesized amide-based mono- and bi-functional monomers to create hydrogels, which also demonstrated excellent compatibility in vivo and high protein repellency (4.3 ng/cm^2^ by SPR) [49,87]. Further investigations are warranted to determine if these types of molecules can produce dental materials with satisfactory mixing with resin monomers, mechanical properties, and degree of conversion.

## 7. Conclusions

Protein-repellent technology has the potential to decrease the global burden of dental caries. When incorporated into composites alone or with QAC monomers, protein-repellent technology can inhibit the adsorption of proteins onto dental materials and thus slow the formation of biofilm and associated oral diseases. Many research groups have contributed to the understanding of protein adsorption and material design, and it is now time for dental material researchers to make an impact in the clinical setting. A concerted effort should be placed on understanding the long-term effect on the oral microflora with protein-repellent restorations, as it is not ideal to remove “good” bacteria from the oral cavity. New, more design-driven monomers should be explored to enhance the stability and protein repellency of QAC restorations, to allow for prolonged antimicrobial properties. Last, the dental material field should unify in using reliable protein-adsorption protocols, to ensure consistent comparisons across different material platforms.

## References

[1] Sugars and Dental Caries. https://www.who.int/news-room/fact-sheets/detail/sugars-and-dental-caries.

[2] Venhoven B.A.M., de Gee A.J., Davidson C.L. (1993). Polymerization contraction and conversion of light-curing BisGMA-based methacrylate resins. Biomaterials.

[3] O’Donnell J.N.R., Skrtic D. (2009). Degree of Vinyl Conversion, Polymerization Shrinkage and Stress Development in Experimental Endodontic Composite. J. Biomim. Biomater. Tissue Eng..

[4] Wang J., Liu Y., Yu J., Sun Y., Xie W. (2020). Study of POSS on the Properties of Novel Inorganic Dental Composite Resin. Polymers.

[5] Liu Y., Wu X., Sun Y., Xie W. (2018). POSS Dental Nanocomposite Resin: Synthesis, Shrinkage, Double Bond Conversion, Hardness, and Resistance Properties. Polymers.

[6] Skrtic D., Antonucci J.M., Eanes E.D. (2003). Amorphous calcium phosphate-based bioactive polymeric composites for mineralized tissue regeneration. J. Res. Natl. Inst. Stand. Technol..

[7] Tanaka J., Inoue K., Masamura H., Matsumura K., Najai H., Inoue K. (1993). The Application of Fluorinated Aromatic Dimethacrylates to Experimental Light-cured Radiopaque Composite Resin, Containing Barium-Borosilicate Glass Filler—A Progress in Nonwaterdegradable Properties. Dent. Mater. J..

[8] Arcís R.W., López-Macipe A., Toledano M., Osorio E., Rodríguez-Clemente R., Murtra J., Fanovich M.A., Pascual C.D. (2002). Mechanical properties of visible light-cured resins reinforced with hydroxyapatite for dental restoration. Dent. Mater..

[9] Kuper N.K., van de Sande F.H., Opdam N.J.M., Bronkhorst E.M., de Soet J.J., Cenci M.S., Huysmans M.C.D.J.N.M. (2015). Restoration Materials and Secondary Caries Using an In Vitro Biofilm Model. J. Dent. Res..

[10] Nedeljkovic I., Teughels W., De Munck J., Van Meerbeek B., Van Landuyt K.L. (2015). Is secondary caries with composites a material-based problem?. Dent. Mater..

[11] Imazato S., Torri M., Tsuchitani Y. (1993). Immobilization of an antibacterial component in composite resin. Dent. Jpn..

[12] Imazato S., Torii M., Tsuchitani Y., McCabe J.F., Russell R.R.B. (1994). Incorporation of Bacterial Inhibitor into Resin Composite. J. Dent. Res..

[13] Gottenbos B., van der Mei H.C., Klatter F., Nieuwenhuis P., Busscher H.J. (2002). In vitro and in vivo antimicrobial activity of covalently coupled quaternary ammonium silane coatings on silicone rubber. Biomaterials.

[14] Murata H., Koepsel R.R., Matyjaszewski K., Russell A.J. (2007). Permanent, non-leaching antibacterial surfaces—2: How high density cationic surfaces kill bacterial cells. Biomaterials.

[15] Lu G., Wu D., Fu R. (2007). Studies on the synthesis and antibacterial activities of polymeric quaternary ammonium salts from dimethylaminoethyl methacrylate. React. Funct. Polym..

[16] Jain A., Duvvuri L.S., Farah S., Beyth N., Domb A.J., Khan W. (2014). Antimicrobial Polymers. Adv. Healthc. Mater..

[17] Li F., Weir M.D., Xu H.H.K. (2013). Effects of Quaternary Ammonium Chain Length on Antibacterial Bonding Agents. J. Dent. Res..

[18] Gozzelino G., Lisanti C., Beneventi S. (2013). Quaternary ammonium monomers for UV crosslinked antibacterial surfaces. Colloids Surf. A Physicochem. Eng. Asp..

[19] He J., Söderling E., Österblad M., Vallittu P.K., Lassila L.V.J. (2011). Synthesis of Methacrylate Monomers with Antibacterial Effects Against S. Mutans. Molecules.

[20] Bienek D.R., Giuseppetti A.A., Okeke U.C., Frukhtbeyn S.A., Dupree P.J., Khajotia S.S., Florez F.L.E., Hiers R.D., Skrtic D. (2020). Antimicrobial, biocompatibility, and physicochemical properties of novel adhesive methacrylate dental monomers. J. Bioact. Compat. Polym..

[21] Xue Y., Xiao H., Zhang Y. (2015). Antimicrobial Polymeric Materials with Quaternary Ammonium and Phosphonium Salts. Int. J. Mol. Sci..

[22] Makvandi P., Jamaledin R., Jabbari M., Nikfarjam N., Borzacchiello A. (2018). Antibacterial quaternary ammonium compounds in dental materials: A systematic review. Dent. Mater..

[23] Antonucci J.M., Zeiger D.N., Tang K., Lin-Gibson S., Fowler B.O., Lin N.J. (2012). Synthesis and characterization of dimethacrylates containing quaternary ammonium functionalities for dental applications. Dent. Mater..

[24] Bienek D., Frukhtbeyn S., Giuseppetti A., Okeke U., Skrtic D. (2018). Antimicrobial Monomers for Polymeric Dental Restoratives: Cytotoxicity and Physicochemical Properties. JFB.

[25] Daood U., Parolia A., Elkezza A., Yiu C.K., Abbott P., Matinlinna J.P., Fawzy A.S. (2019). An in vitro study of a novel quaternary ammonium silane endodontic irrigant. Dent. Mater..

[26] Bienek D.R., Giuseppetti A.A., Frukhtbeyn S.A., Hiers R.D., Esteban Florez F.L., Khajotia S.S., Skrtic D. (2019). Physicochemical, Mechanical, and Antimicrobial Properties of Novel Dental Polymers Containing Quaternary Ammonium and Trimethoxysilyl Functionalities. JFB.

[27] Daood U., Matinlinna J.P., Pichika M.R., Mak K.-K., Nagendrababu V., Fawzy A.S. (2020). A quaternary ammonium silane antimicrobial triggers bacterial membrane and biofilm destruction. Sci. Rep..

[28] Makvandi P., Wang C., Zare E.N., Borzacchiello A., Niu L., Tay F.R. (2020). Metal-Based Nanomaterials in Biomedical Applications: Antimicrobial Activity and Cytotoxicity Aspects. Adv. Funct. Mater..

[29] Imazato S. (2009). Bio-active restorative materials with antibacterial effects: New dimension of innovation in restorative dentistry. Dent. Mater. J..

[30] Li F., Weir M.D., Fouad A.F., Xu H.H.K. (2014). Effect of salivary pellicle on antibacterial activity of novel antibacterial dental adhesives using a dental plaque microcosm biofilm model. Dent. Mater..

[31] Ten Cate J.M. (2006). Biofilms, a new approach to the microbiology of dental plaque. Odontology.

[32] Saini R., Saini S., Sharma S. (2011). Biofilm: A dental microbial infection. J. Nat. Sci. Biol. Med..

[33] Hojo K., Nagaoka S., Ohshima T., Maeda N. (2009). Bacterial Interactions in Dental Biofilm Development. J. Dent. Res..

[34] Gibbons R.J., Hay D.I. (1989). Adsorbed Salivary Acidic Proline-rich Proteins Contribute to the Adhesion of Streptococcus mutans JBP to Apatitic Surfaces. J. Dent. Res..

[35] Spencer P., Ye Q., Misra A., Goncalves S.E.P., Laurence J.S. (2014). Proteins, Pathogens, and Failure at the Composite-Tooth Interface. J. Dent. Res..

[36] Seneviratne C.J., Zhang C.F., Samaranayake L.P. (2011). Dental plaque biofim in oral health and disease. Chin. J. Dent. Res..

[37] Castagnola M., Scarano E., Passali G.C., Messana I., Cabras T., Iavarone F., Cintio G.D., Fiorita A., Corso E.D., Paludetti G. (2017). Salivary biomarkers and proteomics: Future diagnostic and clinical utilities. Acta Otorhinolaryngolog. Italica.

[38] McPherson T.B., Lee S.J., Park K., Horbett T.A., Brash J.L. (1995). Analysis of the Prevention of Protein Adsorption by Steric Repulsion Theory. Proteins at Interfaces II.

[39] Müller C., Wald J., Hoth-Hannig W., Umanskaya N., Scholz D., Hannig M., Ziegler C. (2011). Protein adhesion on dental surfaces—A combined surface analytical approach. Anal. Bioanal. Chem..

[40] Tilton R.D., Robertson C.R., Gast A.P. (1991). Manipulation of hydrophobic interactions in protein adsorption. Langmuir.

[41] Chen S., Li L., Zhao C., Zheng J. (2010). Surface hydration: Principles and applications toward low-fouling/nonfouling biomaterials. Polymer.

[42] (1991). KL Prime; G Whitesides Self-assembled organic monolayers: Model systems for studying adsorption of proteins at surfaces. Science.

[43] Ostuni E., Chapman R.G., Holmlin R.E., Takayama S., Whitesides G.M. (2001). A Survey of Structure−Property Relationships of Surfaces that Resist the Adsorption of Protein. Langmuir.

[44] Chapman R.G., Ostuni E., Takayama S., Holmlin R.E., Yan L., Whitesides G.M. (2000). Surveying for Surfaces that Resist the Adsorption of Proteins. J. Am. Chem. Soc..

[45] Holmlin R.E., Chen X., Chapman R.G., Takayama S., Whitesides G.M. (2001). Zwitterionic SAMs that Resist Nonspecific Adsorption of Protein from Aqueous Buffer. Langmuir.

[46] Bernhard C., Roeters S.J., Franz J., Weidner T., Bonn M., Gonella G. (2017). Repelling and ordering: The influence of poly(ethylene glycol) on protein adsorption. Phys. Chem. Chem. Phys..

[47] Baggerman J., Smulders M.M.J., Zuilhof H. (2019). Romantic Surfaces: A Systematic Overview of Stable, Biospecific, and Antifouling Zwitterionic Surfaces. Langmuir.

[48] Jain P., Hung H.-C., Lin X., Ma J., Zhang P., Sun F., Wu K., Jiang S. (2017). Poly(ectoine) Hydrogels Resist Nonspecific Protein Adsorption. Langmuir.

[49] Chou Y.-N., Sun F., Hung H.-C., Jain P., Sinclair A., Zhang P., Bai T., Chang Y., Wen T.-C., Yu Q. (2016). Ultra-low fouling and high antibody loading zwitterionic hydrogel coatings for sensing and detection in complex media. Acta Biomater..

[50] Sabaté del Río J., Henry O.Y.F., Jolly P., Ingber D.E. (2019). An antifouling coating that enables affinity-based electrochemical biosensing in complex biological fluids. Nat. Nanotechnol..

[51] Migliorini E., Weidenhaupt M., Picart C. (2018). Practical guide to characterize biomolecule adsorption on solid surfaces (Review). Biointerphases.

[52] Prabowo B., Purwidyantri A., Liu K.-C. (2018). Surface Plasmon Resonance Optical Sensor: A Review on Light Source Technology. Biosensors.

[53] Li C., Lu D., Deng J., Zhang X., Yang P. (2019). Amyloid-Like Rapid Surface Modification for Antifouling and In-Depth Remineralization of Dentine Tubules to Treat Dental Hypersensitivity. Adv. Mater..

[54] Bhakta S.A., Evans E., Benavidez T.E., Garcia C.D. (2015). Protein adsorption onto nanomaterials for the development of biosensors and analytical devices: A review. Anal. Chim. Acta.

[55] Höök F., Vörös J., Rodahl M., Kurrat R., Böni P., Ramsden J.J., Textor M., Spencer N.D., Tengvall P., Gold J. (2002). A comparative study of protein adsorption on titanium oxide surfaces using in situ ellipsometry, optical waveguide lightmode spectroscopy, and quartz crystal microbalance/dissipation. Colloids Surf. B Biointerfaces.

[56] Ash A., Mulholland F., Burnett G.R., Wilde P.J. (2014). Structural and compositional changes in the salivary pellicle induced upon exposure to SDS and STP. Biofouling.

[57] Xu Z., Coriand L., Loeffler R., Geis-Gerstorfer J., Zhou Y., Scheideler L., Fleischer M., Gehring F.K., Rupp F. (2019). Saliva-coated titanium biosensor detects specific bacterial adhesion and bactericide caused mass loading upon cell death. Biosens. Bioelectron..

[58] Zhang N., Ma J., Melo M.A.S., Weir M.D., Bai Y., Xu H.H.K. (2015). Protein-repellent and antibacterial dental composite to inhibit biofilms and caries. J. Dent..

[59] Kratz F., Grass S., Umanskaya N., Scheibe C., Müller-Renno C., Davoudi N., Hannig M., Ziegler C. (2015). Cleaning of biomaterial surfaces: Protein removal by different solvents. Colloids Surf. B Biointerfaces.

[60] Kwon J.-S., Lee M.-J., Kim J.-Y., Kim D., Ryu J.-H., Jang S., Kim K.-M., Hwang C.-J., Choi S.-H. (2019). Novel anti-biofouling light-curable fluoride varnish containing 2-methacryloyloxyethyl phosphorylcholine to prevent enamel demineralization. Sci. Rep..

[61] Lee M.-J., Kwon J.-S., Kim J.-Y., Ryu J.-H., Seo J.-Y., Jang S., Kim K.-M., Hwang C.-J., Choi S.-H. (2019). Bioactive resin-based composite with surface pre-reacted glass-ionomer filler and zwitterionic material to prevent the formation of multi-species biofilm. Dent. Mater..

[62] Binnig G., Quate C.F., Gerber C. (1986). Atomic Force Microscope. Phys. Rev. Lett..

[63] Chen X., Davies M.C., Roberts C.J., Tendler S.J.B., Williams P.M., Davies J., Dawkes A.C., Edwards J.C. (1997). Recognition of Protein Adsorption onto Polymer Surfaces by Scanning Force Microscopy and Probe−Surface Adhesion Measurements with Protein-Coated Probes. Langmuir.

[64] Cao L., Wu J., Zhang Q., Baras B., Bhadila G., Li Y., Melo M.A.S., Weir M.D., Bai Y., Zhang N. (2019). Novel Protein-Repellent and Antibacterial Resins and Cements to Inhibit Lesions and Protect Teeth. Int. J. Polym. Sci..

[65] Zhang N., Zhang K., Xie X., Dai Z., Zhao Z., Imazato S., Al-Dulaijan Y., Al-Qarni F., Weir M., Reynolds M. (2018). Nanostructured Polymeric Materials with Protein-Repellent and Anti-Caries Properties for Dental Applications. Nanomaterials.

[66] Zhang N., Chen C., Melo M.A., Bai Y.-X., Cheng L., Xu H.H. (2015). A novel protein-repellent dental composite containing 2-methacryloyloxyethyl phosphorylcholine. Int. J. Oral Sci..

[67] Zhang N., Weir M.D., Romberg E., Bai Y., Xu H.H.K. (2015). Development of novel dental adhesive with double benefits of protein-repellent and antibacterial capabilities. Dent. Mater..

[68] Zhang N., Melo M.A.S., Chen C., Liu J., Weir M.D., Bai Y., Xu H.H.K. (2015). Development of a multifunctional adhesive system for prevention of root caries and secondary caries. Dent. Mater..

[69] Zhang N., Zhang K., Melo M., Weir M., Xu D., Bai Y., Xu H. (2017). Effects of Long-Term Water-Aging on Novel Anti-Biofilm and Protein-Repellent Dental Composite. IJMS.

[70] Wang L., Xie X., Imazato S., Weir M.D., Reynolds M.A., Xu H.H.K. (2016). A protein-repellent and antibacterial nanocomposite for Class-V restorations to inhibit periodontitis-related pathogens. Mater. Sci. Eng. C.

[71] Zhang L., Weir M.D., Chow L.C., Antonucci J.M., Chen J., Xu H.H.K. (2016). Novel rechargeable calcium phosphate dental nanocomposite. Dent. Mater..

[72] Al-Qarni F.D., Tay F., Weir M.D., Melo M.A.S., Sun J., Oates T.W., Xie X., Xu H.H.K. (2018). Protein-repelling adhesive resin containing calcium phosphate nanoparticles with repeated ion-recharge and re-releases. J. Dent..

[73] Al-Dulaijan Y.A., Weir M.D., Melo M.A.S., Sun J., Oates T.W., Zhang K., Xu H.H.K. (2018). Protein-repellent nanocomposite with rechargeable calcium and phosphate for long-term ion release. Dent. Mater..

[74] Cao L., Xie X., Wang B., Weir M.D., Oates T.W., Xu H.H.K., Zhang N., Bai Y. (2018). Protein-repellent and antibacterial effects of a novel polymethyl methacrylate resin. J. Dent..

[75] Choi W., Jin J., Park S., Kim J.-Y., Lee M.-J., Sun H., Kwon J.-S., Lee H., Choi S.-H., Hong J. (2020). Quantitative Interpretation of Hydration Dynamics Enabled the Fabrication of a Zwitterionic Antifouling Surface. ACS Appl. Mater. Interfaces.

[76] Wang L., Xie X., Qi M., Weir M.D., Reynolds M.A., Li C., Zhou C., Xu H.H.K. (2019). Effects of single species versus multispecies periodontal biofilms on the antibacterial efficacy of a novel bioactive Class-V nanocomposite. Dent. Mater..

[77] Chen H., Tang Y., Weir M.D., Lei L., Masri R., Lynch C.D., Oates T.W., Zhang K., Hu T., Xu H.H.K. (2019). Effects of *S. mutans* gene-modification and antibacterial calcium phosphate nanocomposite on secondary caries and marginal enamel hardness. RSC Adv..

[78] Wang H., Wang S., Cheng L., Jiang Y., Melo M.A.S., Weir M.D., Oates T.W., Zhou X., Xu H.H.K. (2019). Novel dental composite with capability to suppress cariogenic species and promote non-cariogenic species in oral biofilms. Mater. Sci. Eng. C.

[79] Fujiwara N., Yumoto H., Miyamoto K., Hirota K., Nakae H., Tanaka S., Murakami K., Kudo Y., Ozaki K., Miyake Y. (2019). 2-Methacryloyloxyethyl phosphorylcholine (MPC)-polymer suppresses an increase of oral bacteria: A single-blind, crossover clinical trial. Clin. Oral Investig..

[80] Li B., Jain P., Ma J., Smith J.K., Yuan Z., Hung H.-C., He Y., Lin X., Wu K., Pfaendtner J. (2019). Trimethylamine N-oxide–derived zwitterionic polymers: A new class of ultralow fouling bioinspired materials. Sci. Adv..

[81] Gonzalez-Bonet A., Kaufman G., Yang Y., Wong C., Jackson A., Huyang G., Bowen R., Sun J. (2015). Preparation of Dental Resins Resistant to Enzymatic and Hydrolytic Degradation in Oral Environments. Biomacromolecules.

[82] Tone S., Hasegawa M., Puppulin L., Pezzotti G., Sudo A. (2018). Surface modifications and oxidative degradation in MPC-grafted highly cross-linked polyethylene liners retrieved from short-term total hip arthroplasty. Acta Biomater..

[83] Van Andel E., Lange S.C., Pujari S.P., Tijhaar E.J., Smulders M.M.J., Savelkoul H.F.J., Zuilhof H. (2019). Systematic Comparison of Zwitterionic and Non-Zwitterionic Antifouling Polymer Brushes on a Bead-Based Platform. Langmuir.

[84] Gu M., Vegas A.J., Anderson D.G., Langer R.S., Kilduff J.E., Belfort G. (2013). Combinatorial synthesis with high throughput discovery of protein-resistant membrane surfaces. Biomaterials.

[85] Imbrogno J., Williams M.D., Belfort G. (2015). A New Combinatorial Method for Synthesizing, Screening, and Discovering Antifouling Surface Chemistries. ACS Appl. Mater. Interfaces.

[86] Li Q., Imbrogno J., Belfort G., Wang X.-L. (2015). Making polymeric membranes antifouling via “grafting from” polymerization of zwitterions. J. Appl. Polym. Sci..

[87] Zhang P., Sun F., Tsao C., Liu S., Jain P., Sinclair A., Hung H.-C., Bai T., Wu K., Jiang S. (2015). Zwitterionic gel encapsulation promotes protein stability, enhances pharmacokinetics, and reduces immunogenicity. Proc. Natl. Acad. Sci. USA.

